# Primary Retroperitoneal Hydatid Cyst: A Diagnostic and Treatment Conundrum

**DOI:** 10.7759/cureus.53842

**Published:** 2024-02-08

**Authors:** Iulian M Slavu, Valeriu Gheorghita, Anca Monica Macovei Oprescu, Florin Filipoiu, Octavian Munteanu, Raluca Tulin, Iulian A Dogaru, Bogdan M Ursuț, Adrian Tulin

**Affiliations:** 1 Anatomy, Carol Davila University of Medicine and Pharmacy, Bucharest, ROU; 2 Infectious Disease, Agrippa Ionescu Emergency Clinical Hospital, Bucharest, ROU; 3 Gastroenterology, Agrippa Ionescu Emergency Clinical Hospital, Bucharest, ROU; 4 Obstetrics and Gynaecology, Carol Davila University of Medicine and Pharmacy, Bucharest, ROU; 5 Anatomy and Embryology, Carol Davila University of Medicine and Pharmacy, Bucharest, ROU; 6 Endocrinology, Agrippa Ionescu Emergency Clinical Hospital, Bucharest, ROU; 7 General Surgery, Agrippa Ionescu Emergency Clinical Hospital, Bucharest, ROU; 8 Medicine, Carol Davila University of Medicine and Pharmacy, Bucharest, ROU

**Keywords:** parasitology, hydatid disease, laparoscopy, infectious, surgery

## Abstract

Hydatid cysts are caused by accidental egg ingestion of the *Echinococcus granulosus* parasite. A 24-year-old female was admitted to our hospital for chronic left lumbar pain. Computed tomography (CT) and abdominal ultrasonography identified an 8/12 cm retroperitoneal cyst. The CT results coupled with enzyme-linked immunosorbent assay tests (positive IgG for *Echinococcus granulosus*) confirmed that the tumor was a hydatid cyst. Treatment consisted of preoperative chemotherapy with albendazole, intraoperative parasite inactivation, laparoscopic partial cystectomy, and drainage. The drain was removed after three days. Chemotherapy was maintained for two years after surgery. No relapse was observed at the six-month reevaluation. In this article, the diagnostic and therapeutic options and resources are discussed and compared with the published literature.

## Introduction

Humans represent an inadvertent host in the infection with the parasite *Echinococcus granulosus*. The incidence can be as high as 1 case per 100,000/inhabitants per year in endemic regions [1,2]. The adult parasite is usually found in the small intestine of dogs. The eggs are passed through feces on the ground/grass [3]. Dogs and other carnivorous animals represent the primary host. Humans are accidental hosts and acquire the infection through inadvertent ingestion of eggs. The larvae hatch from the eggs in the duodenum due to the alkaline environment and pass through the portal system in the liver [3]. On rare occasions, the larvae can bypass the liver and reach the lungs, brain, or any other organ. Once the target organ is reached, the larvae evolve and form vesicular cysts which grow slowly but can reach impressionable sizes. These cysts become symptomatic either through complications of their own or due to compression on neighboring structures. Primary retroperitoneal location of hydatid cysts is rare with a reported incidence in the literature of only 0.5% in endemic regions [4-6]. The mechanism of dissemination in the retroperitoneum is not entirely understood. It is stipulated that the parasite travels through the lymphatic system or systemic circulation to reach the retroperitoneum from the digestive tract [7]. Secondary peritoneal hydatidosis evolves from ruptured liver cysts. The dissemination occurs through larvae contained in the cysts which are called protoscolices [8]. These ruptures can lead to life-threatening immune reactions from the host such as anaphylaxis [8]. Preoperative diagnosis of primary retroperitoneal hydatid cysts is difficult and a high index of suspicion is required. The surgeon needs to know that the lesion is a hydatid cyst and not a tumor as the treatment varies greatly and accidental puncture can lead to further complications. The scope of the article is to bring to attention the fact that hydatidosis should not be ignored as a differential diagnosis in retroperitoneal tumors and explore the diagnostic and therapeutic tools available offering as an example a case treated in our clinic.

## Case presentation

**Figure 1:**
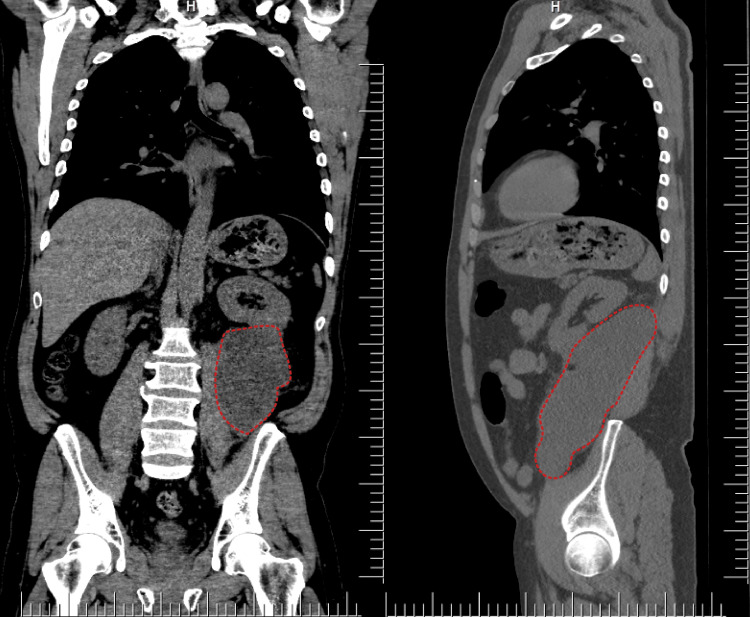
Abdominal CT image with contrast. Left image: sagittal view; right image: coronal view. Red dotted lines represent the retroperitoneal hydatid cyst.

A 24-year-old female patient from a rural area was admitted for chronic left lumbar pain. The symptoms had started a year before but had grown in intensity until presentation at the hospital. The patient lived in the countryside and had daily contact with dogs, sheep, and other animals. On clinical examination, there was fullness on percussion in the left lower abdomen, both anterior and posterior. On palpation, an abdominal tumor was identified, fixed to the surrounding structures, painless with a smooth surface. It was evident at bimanual palpation. An abdominal ultrasound showed a large cystic cavity with a well-defined thick wall, without invasion but with compression on the surrounding organs. A chest X-ray was obtained which was normal. Blood work revealed no modifications except for the enzyme-linked immunosorbent assay (ELISA) test that was positive for IgG. An abdominal and pelvic computed tomography (CT) with intravenous (IV) contrast confirmed the diagnosis of an 8/12 cm cystic mass with multiple septa and negative blood flow in the arterial phase (Figure 1).

**Figure 2:**
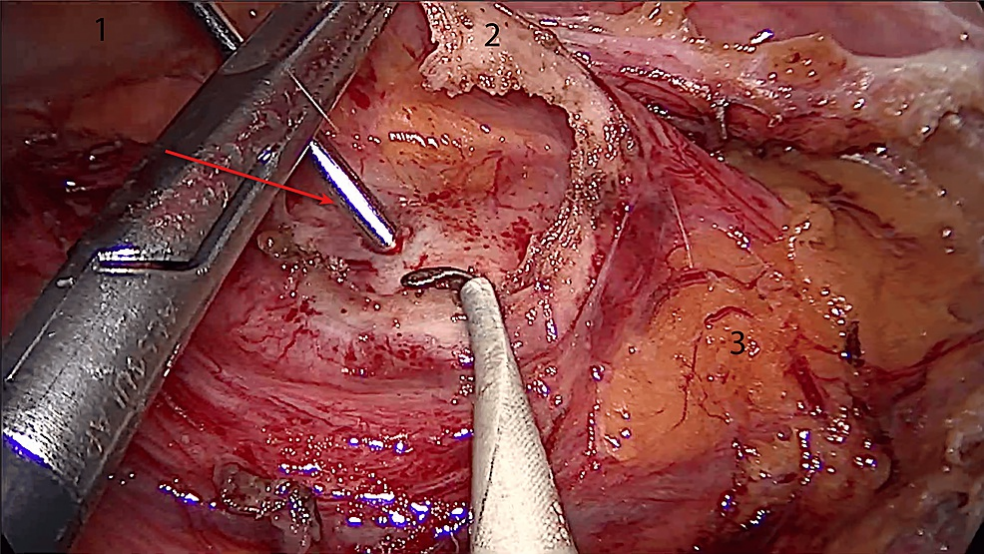
Intraoperative laparoscopic view of the hydatid cyst. 1: inframesocolic posterior parietal peritoneum; 2: hydatid cyst wall; 3: pararenal fat of Gerota. The red arrow points toward the Veres needle which was used to inject the cyst with hypertonic saline.

The diagnosis was confirmed as a primary hydatid retroperitoneal cyst. The patient was started on albendazole at a dose of 400 mg two times/day for one month before surgery. The surgical intervention was done laparoscopically and transperitoneally and consisted of the inactivation of the parasite with scolicidal molecules (Figure 2).

**Figure 3:**
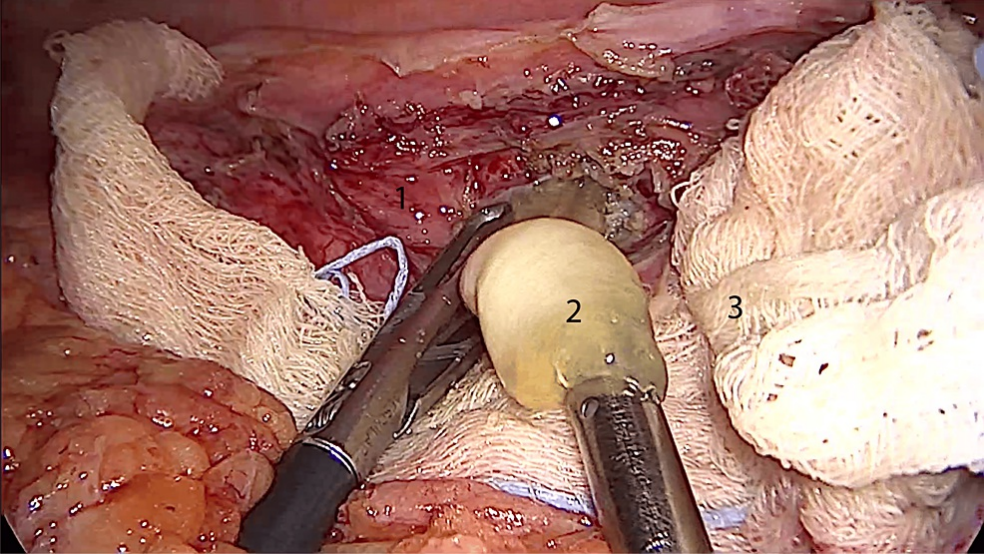
Intraoperative laparoscopic view of the unroofed hydatid cyst. 1: hydatid cyst wall; 2: daughter vesicle; 3: sterile pad soaked in hypertonic saline.

The daughter membranes were extracted (Figure 3).

**Figure 4:**
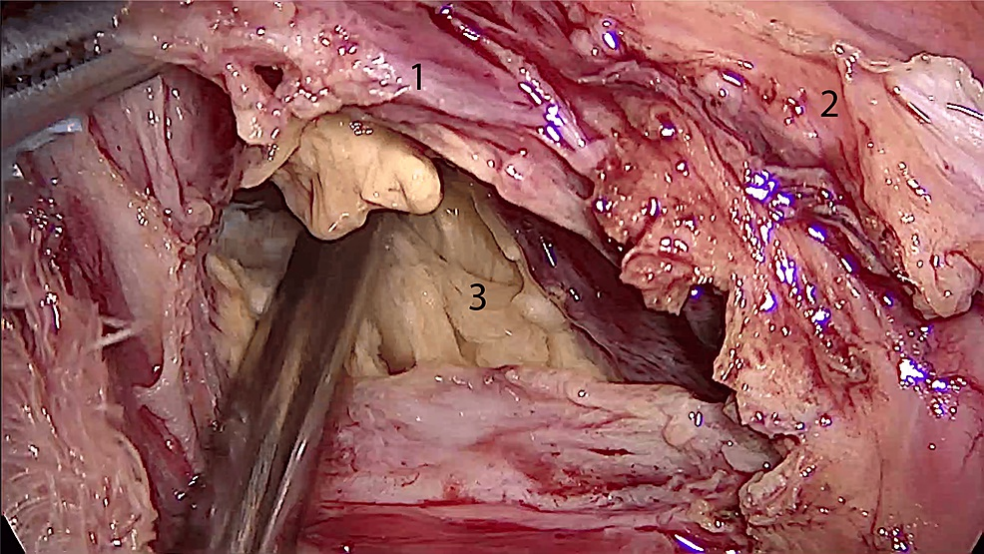
Laparoscopic view of the cyst cavity. 1: posterior parietal peritoneum; 2: hydatid cyst wall; 3: hydatid cyst cavity, emptied.

 A partial cystectomy with unroofing was done to explore the cystic cavity (Figure 4).

**Figure 5:**
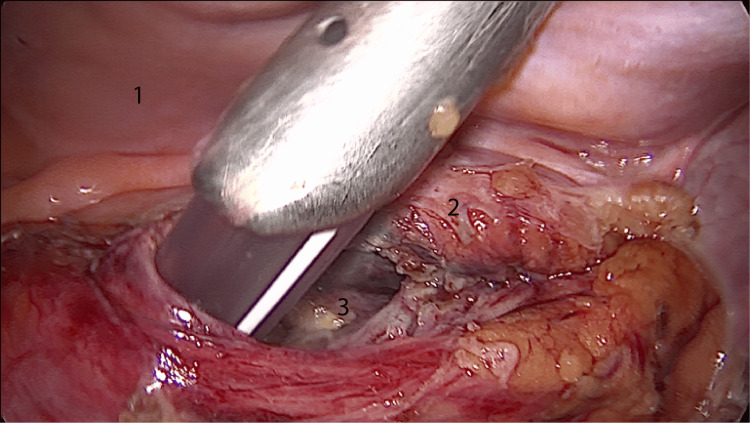
Laparoscopic view of the drainage. 1: left kidney; 2: cavity wall; 3: cystic cavity.

The remaining cavity was drained (Figure 5).

We used hypertonic saline (HS) in concentrations of 20% for five minutes. The content was extracted with an adapted aspiration device. The postoperative period was uneventful. Early ambulation was indicated on day one. The drainage was removed on day three postoperatively when the discharge was below 50 mL. The patient was discharged on day five and continued on albendazole for three months after surgery with regular monthly follow-up evaluations of liver function. At six months, a CT of the abdomen and pelvis with contrast showed no pathological findings. ELISA IgG showed no significant decrease at this point.

## Discussion

Primary retroperitoneal hydatid cysts are rare and difficult entities to diagnose and treat. The largest published database we have identified was reported by Akbulut et al. and involved only 43 cases published in 2010 [9]. From then until 2024, we identified only 30 other case reports published in the PubMed database regarding primary retroperitoneal hydatid cysts.

Hydatidosis treatment has evolved but surgery remains the critical component, and it should be used in conjunction with chemotherapy [10]. Modern techniques, e.g., percutaneous drainage, which were once controversial due to potential complications such as anaphylactic shock or peritoneal dissemination are now used regularly if the size and location of the cyst permits [11].

Hydatidosis represents an important public health issue in endemic countries. It can involve any organ from the brain to muscle tissues and cause significant complications [12,13]. Primary retroperitoneal infections are rare and represent a challenge for the clinician and surgeon as they are asymptomatic until important dimensions of the cyst are reached. The cysts grow at an average of up to 6 cm/year [14]. The first symptoms and those that bring the patient to the hospital are usually related to compression on neighboring organs, as was the case of our patient [15]. The cyst itself may lead to complications such as rupture or infection which can cause anaphylaxis or septicemia [16-18]. Regarding the diagnosis of hydatidosis, blood tests are useful, especially ELISA in endemic areas [19]. As was observed in our case, IgG values for echinococcosis were positive. Ultrasound is always good to use, as it is cheap, readily available, and repeatable. It demonstrates the daughter cysts, floating membranes, and hydatid residue or sand [20]. These elements are encountered only in pathognomonic hydatidosis.

Multiple codification schemes are published by the World Health Organization regarding the ultrasonographic aspect of hydatid cysts [20]. Keep in mind that the radiologist’s closeness to the imaging characteristics is essential for early diagnosis as ultrasonography is operator-dependent. An excellent evaluation is provided by Turgut et al. regarding the different ultrasonography aspects [21]. The gold standard in diagnosis confirmation is CT with IV contrast as it can observe if complications exist, if the cyst is ruptured, if it is active/alive (daughter membranes will exist in it), and if it is inactive (calcification will form in the wall of the cyst) [22]. Either one of these will entail a different line of treatment. If it is active, during surgery, the capsule must not be ruptured before the parasite is inactivated/destroyed with a scolicidal substance such as high-concentration ethanol (concentration above 90%) or HS solution in varying concentrations from 0.09% to 30% [22]. The time until complete destruction of the parasite regarding HS varies from 10 minutes at a concentration of 10% to three minutes at a concentration of 30% [23]. HS can cause diffusion of sodium ions from the peritoneal cavity, which, in turn, can lead to hypernatremia (serum sodium concentration greater than 145 mEq/L). The complications related to hypernatremia are subarachnoid or subdural hemorrhage. The anesthesia team must be informed if HS will be used and when so serum sodium concentration can be observed during and after the surgical intervention. Lavage of the peritoneal cavity in ruptured hydatid cysts with HS does not have a complete scolicidal effect and exposes the patient to dangerous levels of hypernatremia and should not be carried out [23].

Regarding the medical treatment of these cysts, it is mandated for life in the case of inoperable cysts and mandatory after surgery for as long as two years [24]. All inoperable patients should be followed up through regular ultrasonography and CT or magnetic resonance imaging (MRI) at three years to evaluate the disease progression [24]. Albendazole is a good first-line treatment at 10-15 mg/kg daily, divided into two doses [25]. Mebendazole has shown good results with an increased survival rate in up to 80% of the patients treated in comparison to those who did not receive treatment [26]. Both mebendazole and albendazole are parasitostatic not parasitocidal. When administration stops, relapse may occur. The decision to continue or stop administration needs to take into account the viability of the parasite. This is evaluated through antibody values and MRI [27,28]. Complete extraction of the cyst and pericyst when possible is preferred either laparoscopically or through an open technique [29]. Controversies exist regarding the laparoscopic treatment of hydatid cysts due to possible spillage in the peritoneal cavity [30]. If surgeon expertise exists and the cases are properly selected, the results should be as good as the open technique [31].

## Conclusions

In endemic regions, retroperitoneal cysts/tumors merit a differential diagnosis with hydatid cysts. Although ultrasonography can be used as a basic evaluation, to confirm the diagnosis and observe other complications, CT is essential. To decrease relapse, preoperative chemotherapy with albendazole for one month should be followed. Inactivation of the parasite before excision with HS (20% concentration) for two minutes is efficient. Laparoscopy is an adequate method to perform a partial cystectomy of a retroperitoneal hydatid cyst. Management should be multidisciplinary and include discussions with an infectious disease specialist.
